# The association between neighborhood greenness and weight status: an observational study in Perth Western Australia

**DOI:** 10.1186/1476-069X-12-49

**Published:** 2013-06-19

**Authors:** Gavin Pereira, Hayley Christian, Sarah Foster, Bryan J Boruff, Fiona Bull, Matthew Knuiman, Billie Giles-Corti

**Affiliations:** 1 Centre for the Built Environment and Health, School of Population Health, The University of Western Australia, M707, 35 Stirling Highway, Crawley, WA 6009, Australia; 2 Yale Center for Perinatal, Pediatric, and Environmental Epidemiology, Department of Epidemiology and Public Health, School of Medicine, Yale University, New Haven, Connecticut, USA; 3 School of Earth and Environment, The University of Western Australia, Crawley, WA 6009, Australia; 4 McCaughey VicHealth Centre for the Promotion of Mental Health and Community Wellbeing, Melbourne School of Population Health, The University of Melbourne, Melbourne, Victoria, Australia

**Keywords:** BMI, Obesity, Adiposity, NDVI, Vegetation, Built environment

## Abstract

**Background:**

Few studies have examined the relationship between weight status and objectively measured neighborhood greenness and no study has examined this relationship across the different stages of adulthood. This research was an investigation of weight status and neighborhood greenness using objectively measured satellite remote sensing for a large population representative sample.

**Method:**

Cross-sectional study of 10,208 young adults (16–24 years), mid-age adults (25–64 years) and older adults (65+ years) from a population representative sample for the period 2004–2009 in Perth, Western Australia. Neighborhood greenness was ascertained for a 1600m road network service area around each participant’s address using the mean and standard deviation of the Normalized Difference Vegetation Index (NDVI) obtained from remote sensing. Multiple logistic regression was used to assess associations with weight status (overweight-or-obese, obese) adjusted for socio-demographics and health-related behaviors.

**Results:**

The adjusted odds ratio (OR) comparing obesity in the highest to the lowest tertile of mean greenness was 0.78 (95% CI 0.69-0.89). For the same comparison, the OR for overweight-or-obese was similar, 0.84 (95% CI 0.76-0.92). The OR comparing obesity in the highest to lowest tertile of variation in greenness was 0.75 (95% CI 0.66-0.85). For the same comparison, the OR for overweight-or-obese was similar, 0.75 (95% CI 0.68-0.82).

**Conclusion:**

Higher levels and greater variation of neighborhood greenness are associated with lower odds of obesity among adults of all ages. Research examining neighborhood characteristics correlated with variability in greenness will help better understand these relationships.

## Background

Rising levels of obesity are a public health priority [1]. The worldwide prevalence of obesity almost doubled between 1980 and 2008, from 8% to 14% for women and 5% to 10% for men, with disproportionately greater prevalence in high and upper middle income countries [2]. Specifically, the United States and Australian adult populations have experienced marked increases in body mass index (BMI), with Australia’s increasing at approximately 1 kg/m^2^ per decade [1]. Overweight and obesity are important modifiable risk factors for widespread chronic diseases such as cardiovascular disease, hypertension and type II diabetes [3].

Weight status is influenced by a number of factors such as genetic pre-disposition, physical activity and caloric intake [4,5], race or ethnicity [6,7], socioeconomic status [8,9], and social networks [10]. Less well-understood are the potential effects of the built and natural environment [11-14]. Obesity is associated with living in environments where there is poor access to grocery stores, supermarkets and recreation facilities and increased access to fast food outlets and convenience stores [15-18]. Furthermore, some studies have reported an inverse association between neighborhood walkability and body mass index (BMI) among adults [19-22]. However, evidence of the relationship between the natural environment and obesity is less well-established and has mostly been studied in relation to distance and access to parks [23-32] rather than greenness more generally. Moreover, the negative relationship between the amount of green space and levels of BMI has been somewhat consistently reported among children [23,26-30]. However, fewer studies have investigated the influence of green space on levels of BMI among adults and there is insufficient evidence to infer a consistent association [24,25,31,32].

The Normalized Difference Vegetation Index (NDVI) is derived from remote sensing and provides an objective indication of the presence and condition of green vegetation. Historically, the NDVI has provided a method for time series analysis of primary production, phonological patterns and growing season dynamics [33]. The index relies on the relationship between reflected electromagnetic radiation in the red and infrared portions of the spectrum where green healthy vegetation exhibits low red reflectance (due to high chlorophyll absorption) contrasted with high infra reflectance. Although, NDVI has been criticised for instability due to soil and moisture variability, and atmospheric conditions [33], Rhew *et al.*, identified a strong relationship between measures of neighbourhood greenness using NDVI and experts perception of greenness [34].

An inverse relation has been observed between neighborhood greenness as measured by the NDVI and risk of hospitalization for coronary heart disease and stroke [35]. Protective associations remained after adjustment for BMI, implying cardiovascular health benefits of greenness unrelated to intermediate effects on weight status. It remains to be determined whether greenness might also affect weight status and thereby, by association, the array of weight-related morbidities already well-established [3].

The aim of this study was to investigate the association between objectively measured neighborhood greenness (using remote sensing) and weight status (overweight and obese) at different stages of adulthood.

## Methods

### Study design and participants

This was a cross-sectional study of 10,208 participants, consisting of 1,073 young adults (16–24 years), 6,328 mid-age adults (25–64 years) and 2,897 older adults (65+ years) who completed the Western Australian Health and Wellbeing Survey between 2004 and 2009 and who were residents of the Perth metropolitan area. This computer-assisted telephone interview [36] was administered by the Western Australian Department of Health and responses were obtained for a stratified random sample of the state population (N = 1,959,088; 2006 Census). In this study, the Health and Wellbeing Survey was completed once for each participant during the study period (2004–2009). Recruitment and completion of the survey was administered on a monthly basis. The response rate for the Health and Wellbeing Survey is approximately 92% after excluding non-contacts and 76% including non-contacts [36].

### Outcome variables

Self-reported height and weight was obtained from the Health and Wellbeing Survey and used to calculate BMI. Participants aged 18 years and over were classified as *obese* if their BMI (weight (kg)/height (meters)^2^) exceeded 30, and as *overweight-or-obese* if the BMI was above 25. Age and sex specific overweight and obesity cut-offs were used to classify other participants in the young adult age group (16 and 17 year-olds) [37].

### Greenness

Greenness was measured using the Normalized Difference Vegetation Index (NDVI). NDVI provides an indication of the presence and condition of green vegetation with values typically ranging from −1 to +1. Values of −1 generally represent water, while values close to zero (−0.1 to 0.1) correspond to bare surfaces such as rock, sand, rooftops and roads. Higher values (0.2 to 0.4) represent grassland or bush land and values of +1 represent healthy green vegetation [38]. Water features were removed before the NDVI was calculated.

The NDVI was obtained from Landsat imagery, sourced through Landgate’s (Western Australia’s state geospatial data provider) Land Monitor program [39]. The program provides calibrated, cloud free Landsat Thematic Mapper imagery for the south western portion of the state. We obtained Land Monitor distributed imagery that was collected in the summer (January-February) of each year that the health survey was administered in this study (2004–2009). The NDVI assigned to individuals was based on the imagery obtained for the same calendar year as completion of the health survey. We did not have more detailed information on the date of the interview. Each image was ortho-rectified correcting for geometric errors and radiometrically corrected through a calibration routine using a single base scene from 1994 (personal communication, Commonwealth Scientific and Industrial Research Organisation, February 2013). This approach allows for both positional and spectral consistency of NDVI measurements throughout our study.

The resolution of NDVI used in this this study was 30 m × 30 m pixels. The mean of NDVI values (mean greenness) and standard deviation of NDVI values (variation in greenness) were calculated for the 1600 m road network service area around each participant’s address. Neighborhoods around participants’ homes were defined using 1600 m (network distance) service areas based on the assumption that physical activity is the most likely pathway by which neighborhood greenness is negatively associated with BMI. A 1600 m service area represents how far a participant could walk a return trip from home at a moderate to vigorous intensity pace within 30 minutes, which is the recommended level of daily physical activity for adults [40]. A 1600 m service area has been shown to be a critical distance for examining the relationship between parks and walking [41].

The variation in greenness within the service area is influenced by both anthropogenic and natural features. High levels of variability in greenness characterize *mixed use* which occurs in neighborhoods where both high NDVI pixels (green vegetation such as parks, vegetation alongside streets) and low NDVI pixels (non-green destinations such as building roof tops and roads to these destinations) occur together.

Mean greenness and variation in greenness were then classified into tertile groups for analysis based on rationale previously published elsewhere [35]. Briefly, tertiles provided a compromise between capturing the pattern of the association and ensuring sufficient data within each category. Tertiles provided an equal amount of data in each category. As the NDVI is a unitless index of greenness, assessment as a continuous variable for effects per unit increase in the index would be less interpretable than tertiles, which allow interpretation relative to “low”, “medium” and “high” values. Finally, use of tertiles allowed direct comparison of effect estimates with results from a previous study for which associations were observed between greenness and cardiovascular morbidity [35].

### Statistical analysis

Adjustments were made for a range of risk and protective factors for overweight/obesity: age, sex, education, daily serves of fruit and vegetables, and smoking (never versus ever smoked). A complete-case analyses was undertaken due to the very low proportion of participants (0.64%) with a missing value for at least one of the study variables. Multiple logistic regression was used to examine the (adjusted) associations between tertiles for mean greenness and variation in greenness and overweight/obesity. Variance Inflation Factors (VIF) were computed for tertiles of mean greenness and variation in greenness to ascertain the level of collinearity with adjustment variables. Analyses were conducted using SAS v9.2. Weight status, NDVI and sociodemographic data were obtained and processed in 2011 and statistical analyses conducted in 2012.

### Ethics

Approval was obtained from the Human Research Ethics Committees of the Western Australian Department of Health and The University of Western Australia (#2010/1).

## Results

A total of 1,912 (19%) participants were classified as obese and 5,459 (53%) as overweight-or-obese (Table 1). Overall, 7% of young adults (16–24 years), 21% of mid-age adults (25–64 years) and 18% of older adults (65+ years) were obese and the corresponding proportions for overweight-or-obese were 25%, 57% and 56% respectively.

**Table 1:** Distributions of risk and protective factors for obesity among 10,208 adults resident in Perth, Western Australia who responded to the Health and Wellbeing Survey 2004-2009

	**Total**	**Young adults 16–24 years**	**Mid-age adults 25–64 years**	**Older adults 65+ years**
	N=10,208	N=1,073	N=6,238	N=2,897
	**N (%)**	**N (%)**	**N (%)**	**N (%)**
*Weight status*				
Overweight-or-obese	5,459 (53)	263 (25)	3,561 (57)	1,635 (56)
Obese	1,912 (19)	74 (7)	1,304 (21)	534 (18)
*Sex*				
Male	4,318 (42)	506 (47)	2,528 (41)	1,284 (44)
	**Mean (SD)**	**Mean (SD)**	**Mean (SD)**	**Mean (SD)**
*Age*	52 (19)	19 (2)	48 (11)	74 (7)
	**N (%)**	**N (%)**	**N (%)**	**N (%)**
*Highest attained level of education*				
Less than year 10	819 (8)	26 (2)	224 (4)	569 (20)
Year 10 or 11	2,050 (20)	265 (25)	1,151 (18)	634 (22)
Year 12	1,367 (13)	428 (40)	707 (11)	232 (8)
Trade qualification	3,682 (36)	236 (22)	2,393 (38)	1,053 (37)
Tertiary degree	2,254 (22)	118 (11)	1,761 (28)	375 (13)
*Number of serves of vegetables per day*				
Less than one serve	520 (5)	67 (1)	311 (5)	142 (5)
One serve	1,300 (13)	250 (23)	789 (13)	318 (11)
Two serves	2,417 (24)	292 (27)	1,484 (24)	641 (22)
Three serves	2,389 (23)	241 (23)	1,486 (24)	662 (23)
Four serves	1,940 (19)	171 (16)	1,125 (18)	644 (22)
Five serves	1,074 (11)	69 (6)	662 (10)	343 (12)
Six or more serves	546 (5)	38 (4)	372 (6)	136 (5)
*Number of serves of fruit per day*				
Less than one serve	1,721 (17)	226 (21)	1,148 (18)	347 (12)
One serve	2,789 (27)	331 (31)	1,758 (28)	700 (24)
Two serves	3,451 (34)	303 (28)	2,049 (33)	1,099 (38)
Three serves	1,546 (15)	144 (13)	866 (14)	536 (19)
Four or more serves	691 (7)	68 (6)	412 (7)	211 (7)
*Smoking*				
Current smoker	1,435 (14)	137 (13)	1,083 (17)	215 (7)
	**Median (Range)**	**Median (Range)**	**Median (Range)**	**Median (Range)**
*Neighborhood greenness*				
Mean greenness	0.080 (0.396)	0.078 (0.318)	0.079 (0.396)	0.082 (0.387)
Variation in greenness	0.103 (0.158)	0.103 (0.121)	0.103 (0.154)	0.103 (0.145)

Mean greenness for the 10,208 participants’ neighborhoods ranged from −0.059 to 0.337 with a median of 0.081. The variation in greenness ranged from 0.048 to 0.205 with a median of 0.103.

### Risk factors for obese and overweight-or-obese among all adults

The odds ratio for being overweight-or-obese (1.60; 95% CI: 1.36 – 1.88) and the odds ratio for being obese (2.16; 95% CI: 1.78 – 2.62) were higher among adults with educational attainment less than year 10 compared with those with a tertiary degree. Per additional serve of fruit, there was insufficient evidence for a decrease in the odds for being overweight-or-obese (OR 0.98; 95% CI: 0.95 – 1.01) and obese (OR 0.98; 95% CI: 0.94 – 1.02). Similarly, per additional serve of vegetables, there was insufficient evidence for a decrease in the odds for being overweight-or-obese (OR 1.00; 95% CI: 0.98 – 1.02) and obese (OR 1.00; 95% CI: 0.97 – 1.03). For each year increase in age the odds for being overweight-or-obese (OR 1.02; 95% CI: 1.01 – 1.02) and obese (OR 1.01; 95% CI: 1.01 – 1.01) both increased. The odds ratio for being overweight-or-obese (1.67; 95% CI: 1.54 – 1.81) was higher among men. There was insufficient evidence for an association between obesity and sex (Men compared to women, OR 0.92; 95% CI: 0.83 – 1.02). Smoking was not associated with being overweight-or-obese (OR 0.95; 95% CI: 0.85 – 1.06) or obese (OR 0.96; 95% CI: 0.83 – 1.11) among this study population.

### Neighborhood greenness and overweight-or-obese

The adjusted odds ratio for being overweight-or-obese was lower (0.84; 95% CI: 0.76 - 0.92) for adults with high levels of mean greenness (highest tertile), compared with those in neighborhoods with low levels of mean greenness (Table 2). The adjusted odds ratio for being overweight-or-obese was also lower (0.75; 95% CI: 0.68 - 0.82) for adults in the highest variation in greenness tertile compared with the lowest tertile. The adjusted odds ratios for high vs. low tertile in each of the three age groups were all smaller than one although some of the confidence intervals included one. The adjusted odds ratios for moderate vs. low mean and variation in greenness were generally, but not always, closer to unity than the corresponding odds ratio for high vs. low greenness. There was insufficient evidence to indicate that the effects of mean greenness and variation in greenness on overweight-or-obese differed across the three age groups (p-value for interaction 0.668 and 0.952 respectively).

**Table 2:** Odds ratios (OR) and 95% confidence intervals (CI) for being classified as overweight-or-obese for differences in neighborhood greenness among 10,208 adults resident in Perth, Western Australia who responded to the Health and Wellbeing Survey 2004-2009

**Population**		**Unadjusted OR (95% CI)**	**Adjusted**^**a **^**OR (95% CI)**
*All adults*	*Mean greenness in 1600 m service area*^*b*^		
*16+ years*	Low	1	1
	Moderate	1.03 (0.93, 1.13)	0.98 (0.89, 1.09)
	High	**0.87 (0.79, 0.96)**	**0.84 (0.76, 0.92)**
	*Variation in greenness in 1600 m service area*^*c*^		
	Low	1	1
	Moderate	**0.83 (0.75, 0.91)**	**0.80 (0.73, 0.89)**
	High	**0.76 (0.69, 0.84)**	**0.75 (0.68, 0.82)**
*Young adults*	*Mean greenness in 1600 m service area*^*b*^		
*16-24 years*	Low	1	1
	Moderate	0.84 (0.60, 1.18)	0.83 (0.58, 1.19)
	High	0.91 (0.65, 1.27)	0.91 (0.64, 1.29)
	*Variation in greenness in 1600 m service area*^*c*^		
	Low	1	1
	Moderate	0.72 (0.51, 1.00)	**0.67 (0.47, 0.95)**
	High	0.72 (0.51, 1.01)	0.73 (0.51, 1.04)
*Mid-age adults*	*Mean greenness in 1600 m service area*^*b*^		
*25-64 years*	Low	1	1
	Moderate	1.08 (0.96, 1.22)	1.03 (0.91, 1.17)
	High	**0.84 (0.74, 0.95)**	**0.82 (0.72, 0.93)**
	*Variation in greenness in 1600 m service area*^*c*^		
	Low	1	1
	Moderate	**0.81 (0.72, 0.92)**	**0.81 (0.71, 0.92)**
	High	**0.74 (0.65, 0.83)**	**0.75 (0.66, 0.85)**
*Older adults*	*Mean greenness in 1600 m service area*^*b*^		
*65+ years*	Low	1	1
	Moderate	0.92 (0.77, 1.11)	0.90 (0.75, 1.09)
	High	0.86 (0.82, 1.03)	0.87 (0.72, 1.05)
	*Variation in greenness in 1600 m service area*^*c*^		
	Low	1	1
	Moderate	0.89 (0.74, 1.07)	0.90 (0.75, 1.09)
	High	**0.79 (0.66, 0.95)**	**0.83 (0.69, 1.00)**

^a^ Risk/protective factors: age, sex, education, fruit and vegetable intake, smoking.

^b^ Tertiles for mean greenness: Low (mean NDVI < 0.066, reference level), Moderate (0.067 ≤ mean NDVI ≤ 0.094), High (mean NDVI > 0.094)

^c^ Tertiles for variation in greenness: Low (SD NDVI < 0.097, reference level), Moderate (0.097 ≤ SD NDVI ≤ 0.109), High (SD NDVI > 0.109).

Bold text indicates statistical significance at the 5% (α) level.

### Neighborhood greenness and obesity

**Figure 1:**
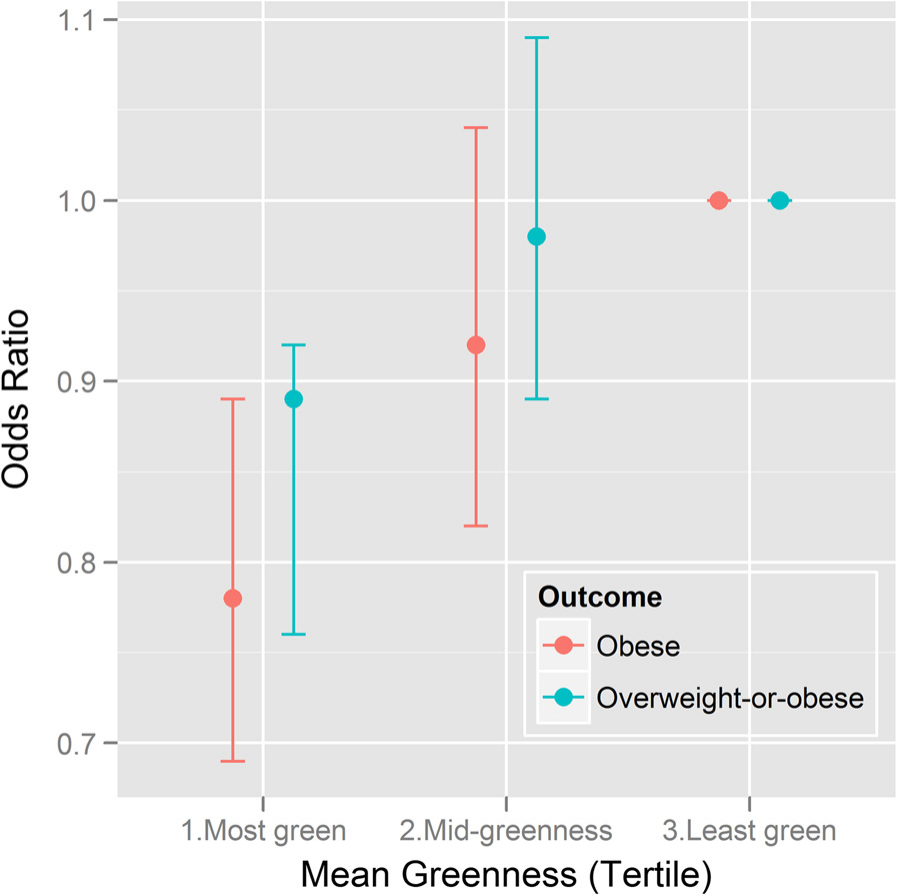
**Adjusted odds ratios and 95% confidence interval bands for being classified as obese and overweight-or-obese for differences in neighborhood mean greenness among 10,208 adults resident in Perth, Western Australia who responded to the Health and Wellbeing Survey 2004–2009.** Effects adjusted for age, sex, education, fruit and vegetable intake, smoking.

**Figure 2:**
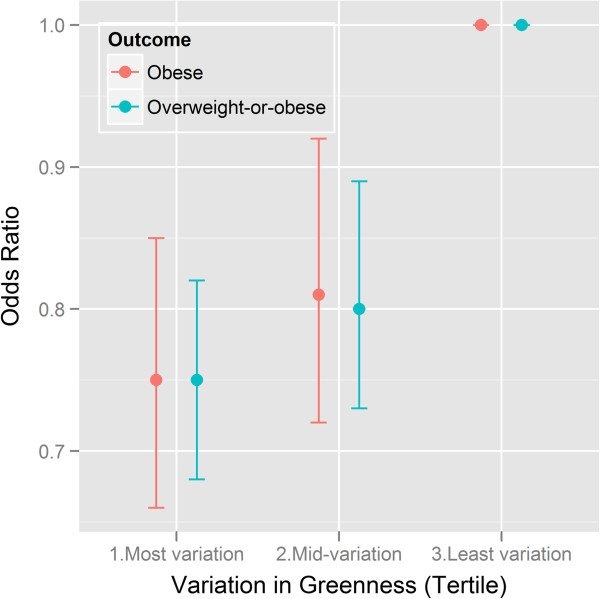
**Adjusted odds ratios and 95% confidence interval bands for being classified as obese and overweight-or-obese for differences in variation in neighborhood greenness among 10,208 adults resident in Perth, Western Australia who responded to the Health and Wellbeing Survey 2004–2009.** Effects adjusted for age, sex, education, fruit and vegetable intake, smoking.

The effect estimates for being obese were largely compatible with those for overweight-or-obese although the effect for mean greenness was stronger for being obese (Figures 1 and 2). The adjusted odds ratio for being obese was lower (0.78; 95% CI: 0.69 - 0.89) for adults with high levels of mean greenness (highest tertile), compared with those in neighborhoods with low levels of mean greenness (Table 3). The adjusted odds ratio for being obese was lower (0.75; 95% CI: 0.66 - 0.85) for adults in the highest variation in greenness tertile compared with the lowest tertile. There was no evidence that the effects of mean greenness and variation in greenness on obesity differed across the three age groups (p-value for interaction 0.920 and 0.672 respectively).

**Table 3:** Odds ratios (OR) and 95% confidence intervals (CI) for being classified as obese for differences in neighborhood greenness among 10,208 adults resident in Perth, Western Australia who responded to the Health and Wellbeing Survey 2004-2009

**Population**		**Unadjusted OR (95% CI)**	**Adjusted**^**a **^**OR (95% CI)**
*All adults*	*Mean greenness in 1600 m service area*^*b*^		
*16+ years*	Low	1	1
	Moderate	0.93 (0.82, 1.05)	0.92 (0.82, 1.04)
	High	**0.77 (0.68, 0.87)**	**0.78 (0.69, 0.89)**
	*Variation in greenness in 1600 m service area*^*c*^		
	Low	1	1
	Moderate	**0.80 (0.71, 0.90)**	**0.81 (0.72, 0.92)**
	High	**0.73 (0.64, 0.82)**	**0.75 (0.66, 0.85)**
*Young adults*	*Mean greenness in 1600 m service area*^*b*^		
*16-24 years*	Low	1	1
	Moderate	**0.40 (0.22, 0.74)**	**0.42 (0.22, 0.78)**
	High	**0.54 (0.31, 0.95)**	0.60 (0.34, 1.06)
	*Variation in greenness in 1600 m service area*^*c*^		
	Low	1	1
	Moderate	0.84 (0.49, 1.45)	0.84 (0.48, 1.47)
	High	0.60 (0.33, 1.09)	0.63 (0.34, 1.17)
*Mid-age adults*	*Mean greenness in 1600 m service area*^*b*^		
*25-64 years*	Low	1	1
	Moderate	1.04 (0.90, 1.21)	1.01 (0.87, 1.17)
	High	**0.82 (0.70, 0.95)**	**0.82 (0.71, 0.96)**
	*Variation in greenness in 1600 m service area*^*c*^		
	Low	1	1
	Moderate	**0.78 (0.68, 0.91)**	**0.80 (0.69, 0.93)**
	High	**0.71 (0.61, 0.82)**	**0.76 (0.66, 0.89)**
*Older adults*	*Mean greenness in 1600 m service area*^*b*^		
*65+ years*	Low	1	1
	Moderate	**0.78 (0.62, 0.98)**	**0.77 (0.61, 0.98)**
	High	**0.69 (0.55, 0.87)**	**0.73 (0.57, 0.92)**
	*Variation in greenness in 1600 m service area*^*c*^		
	Low	1	1
	Moderate	0.86 (0.68, 1.08)	0.91 (0.72, 1.15)
	High	**0.78 (0.62, 0.98)**	0.84 (0.66, 1.07)

^a^ Risk/protective factors: age, sex, education, fruit and vegetable intake, smoking.

^b^ Tertiles for mean greenness: Low (mean NDVI < 0.066, reference level), Moderate (0.067 ≤ mean NDVI ≤ 0.094), High (mean NDVI > 0.094).

^c^ Tertiles for variation in greenness: Low (SD NDVI < 0.097, reference level), Moderate (0.097 ≤ SD NDVI ≤ 0.109), High (SD NDVI > 0.109).

Bold text indicates statistical significance at the 5% (α) level.

The effect of mean greenness was independent of variation in greenness for both the overweight-or-obese and obese classifications of weight status (p-value for interaction 0.729 and 0.964 respectively). For overweight-or-obese, the variation in greenness term was more significant (χ^2^ = 33, p<0.0001) than the mean greenness term (χ^2^ = 12, p=0.0021), when both terms were included in the adjusted model together. Similarly for obesity, when both terms were included in the adjusted model together, the variation in greenness term was more significant (χ^2^ = 21, p<0.0001) than the mean greenness term (χ^2^ = 13, p=0.0018).

### Collinearity between neighborhood greenness and risk factors for adjustment

Variance inflation factors for the measures of greenness across each age group, including the ‘all adults’ group, ranged from 1.31 to 1.42, indicating a low level of collinearity between the exposure of interest in this study and the risk factors used for adjustment.

## Discussion

A negative association was observed between absolute levels (i.e., mean greenness) and variability in neighborhood greenness and obesity. Overall, the odds of obesity was 22% lower for people in high vs. low mean greenness neighborhoods. Similarly, the odds of being overweight-or-obese was 16% lower for people in high vs. low mean greenness neighborhoods. The lower prevalence of obesity among adults in greener areas might be attributable to higher levels of physical activity, such as neighborhood walking, with studies indicating that adults with access to a large high-quality park within walking distance (also 1600 m) from home are more likely to walk, and tend to do so at recommended levels [41,42]. Parks and tree-lined streets are typically representative of green vegetation that might promote physical activity in the neighborhood, as neighborhood attractiveness is consistently associated with increased recreational walking [18]. Associations were observed among young adults, mid-age adults and older adults, which suggests that the protective effect of living in a leafy green neighborhood and variability in greenness on weight status applies throughout adulthood. Although the interaction between stage of adulthood and level of greenness was statistically non-significant, our results stimulate the further hypothesis that associations with weight status might be stronger in early and late adulthood effect as estimates were higher for these age groups. These results might suggest that associations previously reported between greenness and coronary heart disease or stroke might be explained by effects on weight status [35]. Moreover, effects in early adulthood might suggest that greenness and other attributes of the neighborhood built environment might prevent a portion of cardiovascular events or diagnoses that would otherwise be observed later in the life course.

Obesity (and overweight-or-obesity) were more strongly negatively associated with the neighborhood variability in greenness than mean greenness. Overall, there was a 25% lower odds of obesity (and overweight-or-obesity) for those in neighborhoods with high variability in greenness. The interaction between stage of adulthood and variability in greenness was non-significant indicating consistent associations across age groups. A high degree of variability in neighborhood greenness suggests mixed land use, that might, for example, be indicative of neighborhoods that have both a large presence of built destinations and well-connected tree-lined routes to these destinations. A recent review concluded that there is consistent evidence that better access to relevant neighborhood destinations such as local stores and services (which are typically non-green at lower NDVI levels) are conducive for adults’ utilitarian walking [43]. Further studies are required to explore the specific attributes of neighborhoods characterized by high variability in neighborhood greenness. It appears that only one other study has examined the relationship between BMI and greenness in adults using NDVI [44]. In that study, the effect of greenness on BMI was not reported for the study population of 529 adults. However, the authors observed a negative association between BMI and the number of destination types among adults in very green (high NDVI) areas. These results are consistent with our study, in that high levels of variability in greenness analyzed in our study may be comparable to elevated mean greenness combined with the large number of destination types examined in the US study.

Further research is required to identify the mechanisms by which environmental aesthetics [45], mediate engagement in neighborhood activity that might modify weight status. In terms of health more broadly, Hale *et al.* (2011)*,* summarize the challenge of design as the need to create “places aimed at fostering aesthetic experiences that connect individuals to places that support and sustain healthy behaviors” [46]. In this sense, protective associations with weight status observed in this study and previously with cardiovascular disease [35] might have been initially indirectly promoted by unmeasured neighborhood aesthetics and social environments (correlated with variation in greenness) and additionally by experiencing others engaged in health-promoting behaviors in the neighborhood. Alternatively, variation in greenness and its physical environmental correlates might not influence health-related behavior (e.g., physical activity) but be a proxy for the types of neighborhoods within which behavior (e.g., exercise) is socially guided. Individual-based studies are required to further elucidate the pathways by which environmental aesthetics can influence health-related behaviors in the neighborhood environment. Previous studies have shown that perception of the environment is more correlated with behavior (e.g., physical activity) than more objective measures of the environment [47]. Thus, there is a need to identify the aesthetics associated with variability in greenness (e.g., amenities that complement vegetation) that improve positive perception of the neighborhood. Indeed, although greenness as measured by NDVI is an objective measure, the measures of greenness used in this study contain no information on ‘quality’ (e.g. utility or attractiveness of the environment), known to influence behavior [48].

It is possible that the results of this cross-sectional study might be explained by self-selection of individuals of lower-BMI into neighborhoods with both high levels of greenness and variability in greenness. Although adjustment was made for level of education we cannot discount the possibility of residual confounding by socioeconomic status. A further limitation of this study was the absence of specific information on the level of outdoor physical activity undertaken within the neighborhood, a constraint imposed by the use of data from the established survey items in the state health surveillance system.

We note that satellite remote-sensed NDVI is not without imprecision, partially due to cloud cover that limits visibility. This imprecision was minimized via use of composite imagery taken over multiple days, and by taking the imagery in summer when chance of cloud cover is minimal in Perth, Western Australia. This approach also addresses the seasonal fluctuations in NDVI had the imagery been taken throughout the year. We also used NDVI measurements taken each year to account for longer-term changes. However, as this was a cross-sectional study, weight status was only obtained from the health survey at one time for each participant and therefore we could not establish temporal relevance of the association. Although the NDVI is measured with a degree of error, even if the error variance is non-negligible, we have no reason to believe that this error would be directionally associated with weight status. Therefore, the observed effects are likely to be unbiased albeit under-estimated.

## Conclusions

Greater levels of neighborhood greenness and variability in neighborhood greenness are associated with lower odds of obesity among adults. Future natural experiments and cohort studies investigating the effect of natural and built environment attributes on health should include a measure of neighborhood greenness and a measure of variability in neighborhood greenness. Further research is required to examine neighborhood characteristics that correlate with variability in greenness in order to better understand the association between greenness and weight status.

## Abbreviations

NDVI: Normalized difference vegetation index; OR: Odds ratio; CI: Confidence interval; BMI: Body mass index; VIF: Variance inflation factor.

## Competing interests

The authors declare that they have no competing interests.

## Authors’ contributions

GP conceived the greenness and obesity project, conducted the statistical analyses and drafted the manuscript. BGC, MK and HC designed and secured funding for this linked data study. BJB, FCB and BGC designed and led development of the GIS measurements and all authors were involved in redrafting of the manuscript, provided advice on re-analyses for successive drafts, and interpretation of results. All authors read and approved the final manuscript.
